# Superstructure reflections in tilted perovskites

**DOI:** 10.1107/S2053273324007113

**Published:** 2024-07-26

**Authors:** Richard Beanland, Robin Sjökvist

**Affiliations:** a Department of Physics, University of Warwick, Coventry CV4 7AL, UK; Helmholtz Centre for Infection Research, Germany

**Keywords:** perovskites, electron diffraction, structure factors, octahedral tilting

## Abstract

The superstructure spots that appear in diffraction patterns of tilted perovskites are well documented and easily calculated using crystallographic software. Here, by considering a distortion mode as a perturbation of the prototype perovskite structure, it is shown how the structure-factor equation yields Boolean conditions for the presence of superstructure reflections. This approach may have some advantages for the analysis of electron diffraction patterns of perovskites.

## Abstract 2

Boolean conditions are derived from the structure-factor equation, relating distortions of a prototype perovskite structure to the emergence of superstructure reflections.

## 1. Introduction

Conditions for the appearance of superstructure reflections in diffraction from *AB*O_3_ perovskites with *B*O_6_ octahedral tilting were outlined almost exactly 50 years ago in the seminal work of Glazer (1972 ▸, 1975 ▸). In X-ray and neutron diffraction, where many diffracted beams are routinely collected over a wide range of crystal orientations, these reflections, and changes in unit-cell dimensions, may be used to determine the space group, extinction conditions and thus the crystal structure. However, in transmission electron microscopy (TEM) and electron diffraction (ED), excluding 3D-ED methods (Gemmi *et al.*, 2019 ▸), it is usual to examine a few low-index zone axes in individual crystals or domains, which provides only partial data. Nevertheless, this can provide crucial information that is sufficient to distinguish between alternative structures (Woodward & Reaney, 2005 ▸). In addition, the sensitivity of electron scattering to low atomic number (*i.e.* oxygen) atoms and the ability to probe nanoscale regions gives ED an important role in the characterization of perovskite oxides.

With incomplete knowledge of the 3D reciprocal lattice and space group, in ED it is common to work in the reference frame of the prototype perovskite structure while using the term ‘pseudo-cubic’ to acknowledge that this is not actually a correct description of the structure. In the pseudo-cubic reference frame, superstructure reflections that result from larger periods in direct space appear at fractional coordinates in reciprocal space, *i.e.* doubled lattice translations produced by octahedral tilting give reflections at half-order positions. The different patterns of superstructure reflections produced by different Glazer tilt systems have been determined by inspecting simulations (Woodward & Reaney, 2005 ▸) for some low-index ED patterns. Here, we revisit this question and derive general equations for superstructure reflections. The emphasis on tilt system (or other distortion mode), rather than space group, avoids the need to change reference frame according to different choices of unit cell and the conversion of Miller indices describing the zone axis, reciprocal-lattice vectors, and systematic absences this entails. The approach is therefore convenient when analysing diffraction patterns of perovskites exhibiting different distortion modes, and provides a result for any zone axis.

## 2. Calculation

Ignoring thermal factors, the structure-factor equation that gives the complex amplitude of a diffracted beam **g** in a crystal with a static distortion mode can be written 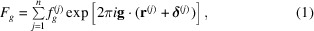
where the sum is taken over all *j* atoms in the unit cell, each having atomic scattering factor 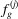, fractional coordinates 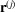 in the prototype structure, and static displacement from these prototype coordinates (due to a distortion mode, such as an oxygen octahedral tilt system) 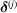.

Here, we are not interested in the precise value of *F*_*g*_ for a superstructure reflection. Rather, our main concern is whether a distortion mode produces a superstructure reflection or not. The answer to this question is simply that a reflection will be present when the result of equation (1) is not exactly zero and, as is shown below, this can be determined most easily by allowing 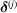 to be arbitrarily small. This approach also means that any second-order effects (*e.g.* octahedral distortions) can be neglected. Using the approximation *e*^(*a*+*b*)^ = *e*^*a*^*e*^*b*^ = *e*^*a*^(1 + *b*) for small *b*, and noting that the structure factor for superstructure reflections in the prototype structure is precisely zero, the structure factor of a superstructure reflection with infinitesimal 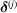 is 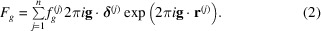
On first sight, equation (2) does not appear to be much more informative than equation (1), but further simplification can be obtained, as follows.

We choose a unit cell that is twice the size of the prototype in all three dimensions, which is large enough to be a unit cell for any Glazer octahedral tilting pattern (although it will not generally correspond to the fundamental unit cell of the distorted structure). In this reference frame, superstructure reflections have integer Miller indices with at least one odd index, while reflections of the prototype structure have all-even indices (*i.e.* integer indices in the pseudo-cubic frame). This expanded cell is eight times the size of the prototype cell and has 24 oxygen atoms with coordinates given in Table 1 ▸. Here, for reasons that will shortly become apparent, we write the oxygen coordinates *r*_*i*_, which all have positions that are multiples of a quarter of the lattice parameter of the unit cell, with an integer form *s*_*i*_ = 4*r*_*i*_. The exponential term can then be written 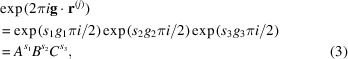
using the substitution 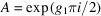, 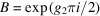 and 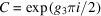. The Miller indices *g*_*i*_ are integers and thus the terms *A*, *B* and *C* take values of ± 1 for even *g*_*i*_ and ± *i* for odd *g*_*i*_.

**Figure 1:**
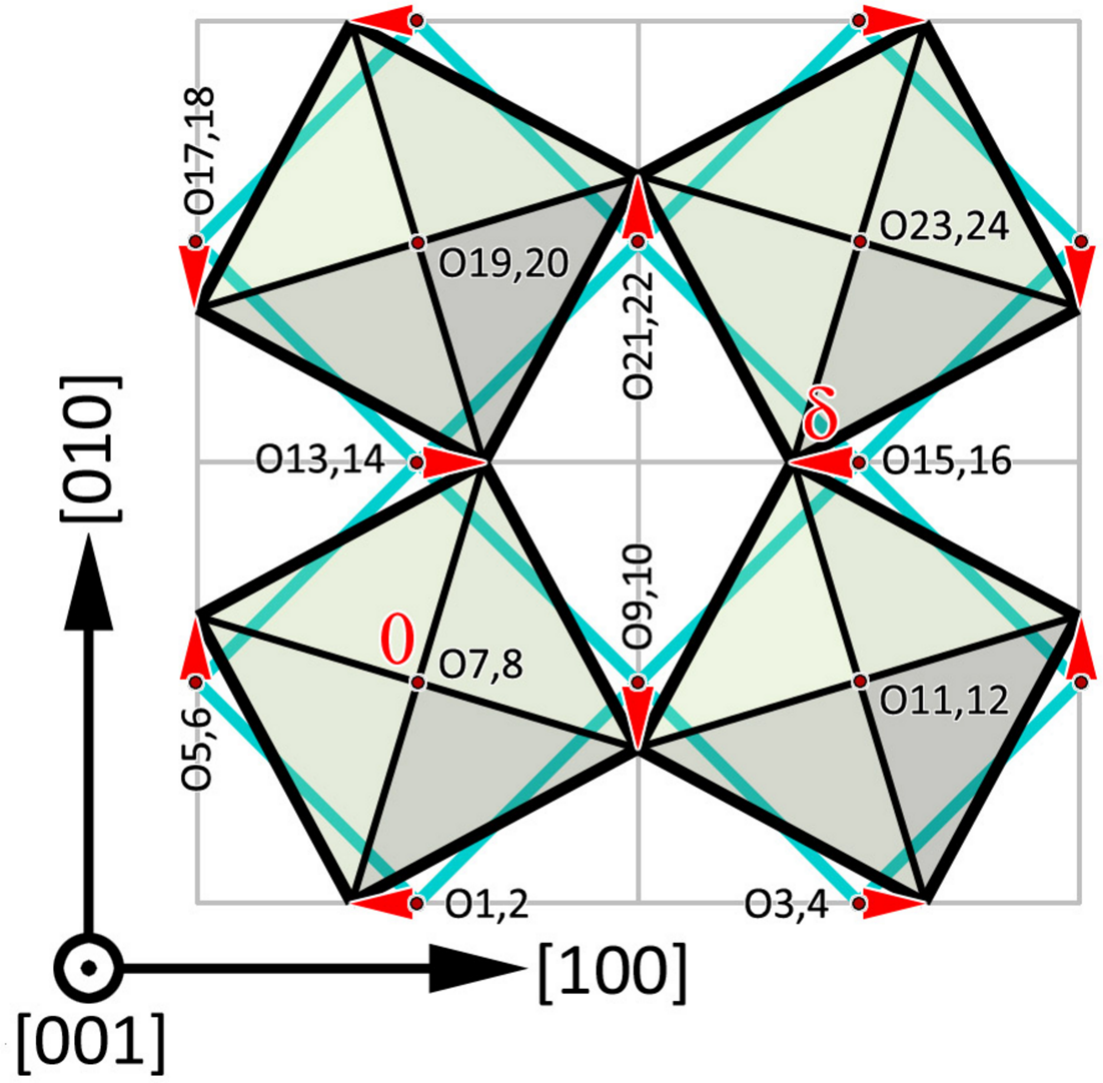
Displacements of oxygen atoms due to a *B*O_6_ octahedral rotation about the *c* axis. Labels correspond to Table 1 ▸; red arrows indicate displacements 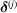 listed in Table 2 ▸.

We are now ready to consider specific distortion modes. Fig. 1 ▸ shows the direction of (infinitesimal) oxygen-atom displacements for in-phase octahedral rotations about *c*, *a*^0^*a*^0^*c*^+^ in Glazer notation, which are listed in Table 2 ▸. Using equation (3) and substituting into equation (2) we obtain 
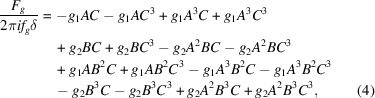
which nicely reduces to 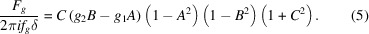
This equation can be interpreted as a set of Boolean conditions, all of which must be satisfied for a superstructure reflection to exist. Thus, since *A* = ±1 for even *g*_1_ and *A* = ±*i* for odd *g*_1_, (1 − *A*^2^) is only non-zero, and a superstructure reflection will only be present, when the first index of the reflection, *g*_1_, is odd. Similarly, (1 + *C*^2^) is only non-zero for even *g*_3_ and therefore equation (5) indicates that superstructure reflections of the *a*^0^*a*^0^*c*^+^ tilt system must have the form odd–odd–even in the frame of the doubled cell. As for the other two terms in equation (5), 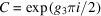 is never zero, while (*g*_2_*B* − *g*_1_*A*) = 0 when |*g*_1_| = |*g*_2_|. We thus obtain the result that superstructure reflections occur with pseudo-cubic indices 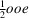, |*g*_1_| ≠ |*g*_2_|. The latter condition describes the systematic absences that result from the *b*-glide plane in the space group of the *P*4/*mbma*^0^*a*^0^*c*^+^ structure.

A similar procedure can be performed for the *a*^0^*a*^0^*c*^−^ tilt system, in which the displacements of oxygen atoms with even-numbered labels in Table 2 ▸ are reversed, with the result 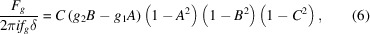
indicating that superstructure reflections must have pseudo-cubic form 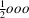 with the same systematic absences, this time from the *c* glide in the space group *I*4/*mcm* of the *a*^0^*a*^0^*c*^−^ structure.

Other distortion modes, such as antiferrodistortive cation displacements, can also be considered in a similar manner using their coordinates and displacements.

A particularly elegant aspect of this approach is that rules for superstructure reflections in structures with oxygen octahedral tilts about multiple axes – or, indeed, multiple distortion modes (*e.g.* antiferrodistortive displacements, distorted oxygen octahedra *etc*.) – can be constructed simply by adding equations, giving a straightforward and quick method of calculation. The results are summarized for the 14 crystallographically distinct oxygen octahedral tilt systems (Howard & Stokes, 1998 ▸) in the Appendix[App appa].

## 3. Conclusions

Equations governing the appearance of superstructure spots resulting from distortion modes in perovskites have been derived. This may aid the interpretation of ED patterns and replicates the work of Glazer (1975 ▸) and Woodward & Reaney (2005 ▸). The emphasis on distortion mode, rather than space group, allows the interpretation of ED patterns without the need to rewrite vectors in real and reciprocal space for different unit cells.

## Figures and Tables

**Table 1:** Coordinates of the 24 oxygen atoms in the expanded unit cell used to describe tilted perovskites (Fig. 1 ▸, written as integer multiples of 1/4, *e.g.*
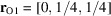

Atom	*s*_1_	*s*_2_	*s*_3_	Atom	*s*_1_	*s*_2_	*s*_3_
O1	1	0	1	O13	1	2	1
O2	1	0	3	O14	1	2	3
O3	3	0	1	O15	3	2	1
O4	3	0	3	O16	3	2	3
O5	0	1	1	O17	0	3	1
O6	0	1	3	O18	0	3	3
O7	1	1	0	O19	1	3	0
O8	1	1	2	O20	3	3	2
O9	2	1	1	O21	2	3	1
O10	2	1	3	O22	2	3	3
O11	3	1	0	O23	3	3	0
O12	3	1	2	O24	3	3	2

**Table 2:** Oxygen-atom displacements produced by the *a*^0^*a*^0^*c*^+^ tilt system shown in Fig. 1 ▸ Displacements in the *a*^0^*a*^0^*c*^−^ tilt system are similar, except O2, O4, O6, O10, O14, O16, O18 and O22 whose displacements are reversed.

Atom	δ_1_	δ_2_	δ_3_	Atom	δ_1_	δ_2_	δ_3_
O1	−δ	0	0	O13	δ	0	0
O2	−δ	0	0	O14	δ	0	0
O3	δ	0	0	O15	−δ	0	0
O4	δ	0	0	O16	−δ	0	0
O5	0	δ	0	O17	0	−δ	0
O6	0	δ	0	O18	0	−δ	0
O7	0	0	0	O19	0	0	0
O8	0	0	0	O20	0	0	0
O9	0	−δ	0	O21	0	δ	0
O10	0	−δ	0	O22	0	δ	0
O11	0	0	0	O23	0	0	0
O12	0	0	0	O24	0	0	0

**Table 3:** Pseudo-cubic superstructure reflections in perovskites with octahedral tilting Miller indices are given in the form 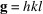 to allow for concise descriptions of extinctions.

No.	Tilt system	Space group	Conditions for superstructure spots to exist
1	*a* ^+^ *a* ^+^ *a* ^+^	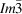	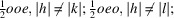 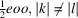
			
2	*a* ^0^ *b* ^+^ *b* ^+^	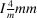	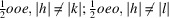
			
3	*a* ^0^ *a* ^0^ *c* ^+^	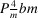	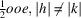
			
4	*a* ^0^ *a* ^0^ *c* ^−^	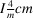	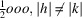
			
5	*a* ^0^ *b* ^−^ *b* ^−^	*Imma*	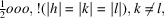 !(*h* = *n*, *k* = ±*n* + 4*m*, *l* = ∓*n* + 4*m*), *n*, *m* integers
			
6	*a* ^−^ *a* ^−^ *a* ^−^	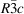	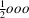 , 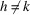 and 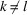 and 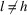 , !(*h* = *n*, *k* = *n* + 4*m*, *l* = *n* + 4*p*), *n*, *m*, *p* integers
			
7	*a* ^+^ *b* ^+^ *c* ^+^	*Immm*	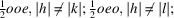 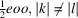
			
8	*a* ^+^ *a* ^+^ *c* ^−^	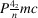	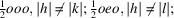 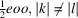
			
9	*a* ^0^ *b* ^+^ *c* ^−^	*Cmcm*	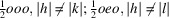
			
10	*a* ^+^ *b* ^−^ *b* ^−^	*Pnma*	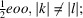 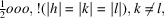 !(*h* = *n*, *k* = ±*n* + 4*m*, *l* = ∓*n* + 4*m*), *n*, *m* integers
			
11	*a* ^0^ *b* ^−^ *c* ^−^	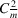	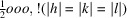
			
12	*a* ^−^ *b* ^−^ *b* ^−^	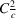	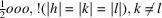
			
13	*a* ^+^ *b* ^−^ *c* ^−^	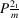	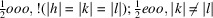
			
14	*a* ^−^ *b* ^−^ *c* ^−^	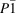	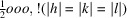

## Appendix A

For completeness we compile in Table 3 ▸ the conditions governing the existence of superstructure spots in the pseudo-cubic reference frame for the 14 crystallographically distinct Glazer tilt systems as listed by Howard & Stokes (1998 ▸). These may be obtained by adding equations similar to equations (5) and/or (6) for the appropriate tilt system.

Some general rules become apparent from Table 3 ▸. The rules for in-phase tilting are quite straightforward, with each tilt system *a*^+^, *b*^+^, *c*^+^ producing its own set of superstructure spots with pseudo-cubic indices , ,  with no dependence on the presence of any other distortions. Conversely, all antiphase tilt systems produce pseudo-cubic  superstructure spots, and are only distinguished by their systematic absences. Furthermore, because in these cases the type of superstructure reflections is always the same, tilts of equal magnitude operating about different axes can result in changes to the set of systematic absences. Accordingly, systematic absences are most apparent for the *a*^−^*a*^−^*a*^−^ system. This means that determining antiphase tilting systems is less straightforward than in-phase tilt systems. For investigations using ED, it may thus be important to explore reciprocal space in 3D since systematic absences can readily be ‘filled in’ by double diffraction where the possibility exists, particularly in the zero-order Laue zone. Access to higher-order Laue zones, or zone axes where no double diffraction pathways are present, is generally necessary.

Table 3 ▸ shows that reflections of the form ,  and  do not result from oxygen octahedral tilting. They may, however, be produced by antiferrodistortive displacements of cations. Calculation of extinction rules for these distortion modes is left as an exercise for the reader.
